# Heterogeneity in malaria exposure and vaccine response: implications for the interpretation of vaccine efficacy trials

**DOI:** 10.1186/1475-2875-9-82

**Published:** 2010-03-23

**Authors:** Michael T White, Jamie T Griffin, Chris J Drakeley, Azra C Ghani

**Affiliations:** 1 MRC Centre for Outbreak Analysis & Modelling, Department of Infectious Disease Epidemiology, Faculty of Medicine, Imperial College London, London, UK; 2 Department of Infectious and Tropical Diseases, London School of Hygiene and Tropical Medicine, London, UK

## Abstract

**Background:**

Phase III trials of the malaria vaccine, RTS, S, are now underway across multiple sites of varying transmission intensity in Africa. Heterogeneity in exposure, vaccine response and waning of efficacy may bias estimates of vaccine efficacy.

**Methods:**

Theoretical arguments are used to identify the expected effects of a) heterogeneity in exposure to infectious bites; b) heterogeneity in individual's response to the vaccine; and c) waning efficacy on measures of vaccine efficacy from clinical trials for an infection-blocking vaccine.

**Results:**

Heterogeneity in exposure and vaccine response leads to a smaller proportion of trial participants becoming infected than one would expect in a homogeneous setting. This causes estimates of vaccine efficacy from clinical trials to be underestimated if transmission heterogeneity is ignored, and overestimated if heterogeneity in vaccine response is ignored. Waning of vaccine efficacy can bias estimates of vaccine efficacy in both directions.

**Conclusions:**

Failure to account for heterogeneities in exposure and response, and waning of efficacy in clinical trials can lead to biased estimates of malaria vaccine efficacy. Appropriate methods to reduce these biases need to be used to ensure accurate interpretation and comparability between trial sites of results from the upcoming Phase III clinical trials of RTS, S.

## Background

Malaria poses a major public health problem with unacceptably high levels of *Plasmodium falciparum*-associated morbidity and mortality recorded worldwide [1]. In recent years, dramatic declines in both parasite prevalence and disease incidence have been observed in a number of locations across Africa [2-4], likely attributable to the increased access to effective first-line therapy plus roll-out of interventions including long-lasting insecticide treated nets. Whilst these may be considered a success, an effective vaccine would be an important addition to the current suite of anti-malaria interventions. The most promising candidate malaria vaccine under development is RTS, S combined with GlaxoSmithKline's AS01 adjuvant.

The principal component of RTS, S is the circumsporozoite antigen, which induces pre-erythrocytic antibodies that prevent sporozoites from infectious mosquito bites from leading to blood-stage malaria. RTS, S is thus an infection-blocking vaccine. Phase II trials of RTS, S/AS have demonstrated significant efficacy against time to first infection (45% (95% CI, 31.4-55.9) [5] and 65.2% (95% CI, 20.7-84.7) [6] reduction), clinical malaria (29.9% (95% CI, 11.0-44.8) [5], 56% (95% CI, 31-72) [7] and 43.2% (95% CI, -47.1-78.0) [6] reduction in different trials) and severe malaria (57.7% (95% CI, 16.2-80.6) [5] reduction). Multi-centre Phase III trials for this vaccine are now underway in 11 sites across Africa.

There are many sources of bias that may arise when measuring an infection-blocking malaria vaccine's efficacy in clinical trials. A significant source of bias that is often overlooked is due to the effects of heterogeneity. Heterogeneity can arise in one of three ways. Firstly, heterogeneity in exposure occurs due to some individuals being bitten more frequently than others. Between-individual variation has been observed in experimental trials in which differential attractiveness to humans sleeping in tents was quantified using mosquito traps [8]. This heterogeneity in biting rate has also been attributed to age due to difference in surface area between adults and children [9], differences in human sweat components [10], proximity to larval breeding sites [11], bed net usage [12] and differences in house design [13].

Secondly, there can be heterogeneity in vaccine response, which occurs due to variation in the degree of protection (or take) of the vaccine between individuals. For example if a vaccine is reported to have 90% efficacy against infection, at one extreme this could mean that each infection will be blocked 90% of the time, whilst at the other extreme it could mean that 90% of vaccinated people will be completely protected from infection. Smith *et al *[14] and Halloran *et al *[15] have investigated this concept and coined the terms all-or-nothing and leaky vaccines. An all-or-nothing vaccine is one which offers complete protection to a subset of the vaccinated population and has no effect on the rest of the population whilst a leaky vaccine is one which offers the same level of partial protection to everyone. In a malaria vaccine trial it is possible that the candidate vaccine will induce a different level of protection in each of the trial participants. The level of protection given to a vaccinated person will be described by their vaccine response, which will depend on the relative immunogenicity of the vaccine and the magnitude of the induced antibody titres. Individual vaccine response may further depend on genetic and nutritional factors [16] as well as age, past exposure and season [17].

Finally, for a candidate malaria vaccine offering partial protection from infection, waning of efficacy is also likely to be observed. Understanding how a vaccine's efficacy wanes over time is at least as important as measuring its initial efficacy. Waning of efficacy can be estimated from clinical trials by measuring a vaccine's half-life: the time taken for the initial efficacy to be halved. However, a vaccine's half-life is a crude measure of waning, as the effects of a vaccine may not decay at a constant rate, and thus some people may remain protected for longer than others. This type of heterogeneity is often neglected in the interpretation of trial results.

Mathematical models have been used to investigate the impact of heterogeneity in exposure [18-20], heterogeneity in vaccine response [20,21] and waning of efficacy [20] on malaria transmission. In this paper theoretical arguments are used to understand how these three types of heterogeneity can affect measurement of vaccine efficacy in clinical trials. Examples are motivated by the RTS, S vaccine and hence focus on a pre-erythrocytic vaccine which is assumed to act by partially blocking infection. In the first section the basic methods currently used to estimate vaccine efficacy in Phase II and Phase III trials are described. The effects of the three types of heterogeneity on the estimates of vaccine efficacy from clinical trials are then investigated.

### Measurements of vaccine efficacy

For a vaccine with efficacy *VE*, if the probability of an unvaccinated person becoming infected after an infectious bite is *b*, then the probability that a vaccinated person becomes infected is (1 - *VE*) *b*. This efficacy *VE*, hereafter referred to as the individual efficacy, can be defined as the proportion of infectious bites on a vaccinated population that are blocked by the vaccine. This proportion can be directly estimated in laboratory based Phase IIa trials where healthy adult volunteers are exposed to bites from infectious mosquitoes and then monitored for blood-stage malaria infection [22,23].

In field-based Phase IIb clinical trials in malaria endemic regions, it is impossible to directly estimate this proportion. Instead vaccine efficacy must be estimated by measuring malaria-related events in both the vaccine and control arms of the trial. Vaccine efficacy can then be calculated as

Malaria infection has a complex life history giving rise to a choice of events to measure [24]. These include prevalence of infection as detected by the presence of parasitaemia, time to first infection, episodes of febrile malaria, episodes of severe malaria or multiple episodes in fixed time periods. Each of these end-points will be relevant in different settings. For example, infection-blocking efficacy would be most important to a traveller to a malaria endemic region, whereas efficacy against severe disease would be most important for a child living in an area of high transmission. Vaccine efficacy against time to first infection and prevalence of infection are focused upon as these are the outcomes directly affected by pre-erythrocytic vaccines, although they can also be expected to have a downstream effect on morbidity and mortality.

Following the notation of Smith *et al *[14], the vaccine efficacy against cumulative incidence of infection, also known as risk-based infection-blocking efficacy, is denoted *VE*_*r*_. This gives a measure of the risk of having become infected by the end of a trial for the vaccine group compared to the control group. Note that the cumulative incidence of infection is different to the prevalence of infection as it measures the proportion who have ever become infected and not the proportion infected at a given time. Vaccine efficacy against time to first infection, also known as rate-based infection-blocking efficacy, will be denoted *VE*_*f*_. This gives a measure of the rate at which the vaccine group becomes infected compared to the control group.

In a trial of a vaccine with individual efficacy *VE*, of length *T*, in a setting with constant force of infection Λ, estimates for the proportion infected and the person years at risk (PYAR) can be calculated using the formulae in Table 1.

**Table 1:** Expected values for the observed infected proportion *I*(*T*), and person years at risk (PYAR) in a clinical trial with follow-up period *T*, of a vaccine with individual efficacy *VE*, in a region with force of infection Λ.

Group	Proportion infected *I*(*T*)	Person years at risk PYAR
Control	1 - *e*^-Λ*T*^	
Vaccine	1 - *e*^-(1 - *VE*)Λ*T*^	

The risk-based efficacy can be calculated as

Thus the risk-based efficacy, *VE*, as measured in a field trial can be estimated from the individual efficacy. This measure approaches 0 as *T *increases as it is assumed that everyone will become infected if the follow-up is sufficiently long. In addition, for small *T *we have *VE*_*r *_≈ *VE*.

In a field trial the rate at which a cohort becomes infected can be estimated by dividing the infected proportion at the end of the follow-up period by the total person years at risk. The rate-based efficacy can then be calculated as

Therefore the rate-based efficacy is equal to the individual efficacy. In particular it is independent of the force of infection Λ and the length of the trial *T*.

The risk-based definition of efficacy is commonly used for diseases in which natural infection with the causative agent provides near complete protection against a second infection with the same agent, for example measles. However, the high frequency at which people living in endemic regions are challenged with malaria infection makes risk-based infection-blocking efficacy uninformative, as most trial participants are likely to have become infected during a trial. In order to overcome this difficulty, a consensus has formed amongst vaccine trialists to primarily use rate-based measures based on time to first infection [24,25].

### Heterogeneity in transmission

In a site where a malaria intervention is being trialled, the transmission intensity is usually measured using the entomological inoculation rate (EIR). In the analysis of trial results it is often assumed that all participants experience the same homogeneous force of infection. Making this assumption and ignoring the effects of heterogeneity in transmission can bias trial results.

If the average force of infection is measured to be Λ = *bε*, where *b *is the infectivity (the probability that a bite from an infectious mosquito results in infection), and *ε *is the measured EIR, then the heterogeneity in malaria transmission can be modelled using some distribution *f*. A proportion *f*(*x*) of the population under observation will experience a force of infection *x*Λ. As *f *is a distribution it must have mean 1 ensuring that that the average force of infection across the entire population is indeed Λ. Examples of distributions that will be considered are given in Table 2.

**Table 2:** Examples of distributions describing heterogeneity in exposure in order of increasing heterogeneity.

Distribution	Description
constant	All individuals receive the same number of infectious mosquito bites.
80/20	The distribution of infectious bites follows an 80/20 rule as suggested by Woolhouse *et al *[28]where 20% of people receive 80% of the bites.
gamma	The distribution of infectious mosquito bites follows a gamma distribution with parameter 1/4.2 as suggested by Smith *et al *[19].
extreme	A hypothetical example of extreme heterogeneity as might be observed in a localised epidemic, in this case modelled as 5% of people receiving 95% of infectious bites.

In an area with constant force of infection Λ (i.e. homogeneous transmission), the average proportion infected at time *T *will be *I*(*T*) = 1-*e*^-Λ*T*^. In an area with heterogeneity in transmission described by a distribution *f*, the average proportion infected will be

**Figure 1:**
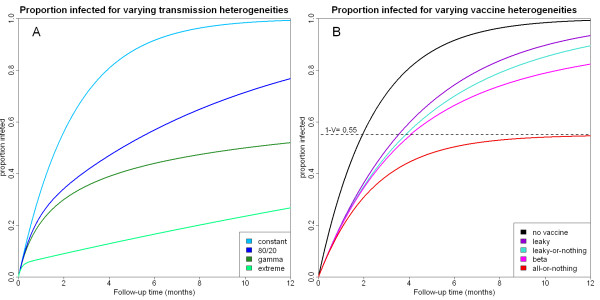
**Cumulative proportion of infected trial participants for an infection-blocking vaccine with an average individual efficacy of 45.0% based on RTS,S, and in a similar setting to the Mozambique trial site described by Alonso *et al ***[5]. (A) Cumulative proportion of unvaccinated trial participants infected under a range of transmission heterogeneities. (B) Cumulative proportion of vaccinated trial participants infected for a range of vaccine types. The proportion infected for an all-or-nothing vaccine can never cross the dashed line marked 1-V = 0.55 as the 45% of vaccinees with total protection will never become infected. Note that the 4 vaccines each have the same individual efficacy but different heterogeneities in vaccine efficacy.

Figure 1A shows the average infected proportion in a trial as a function of the follow-up time *T *for the heterogeneity distributions listed in Table 2. As heterogeneity in exposure to infection increases, the proportion of individuals infected in any given setting decreases. Furthermore, for a given force of infection, homogeneous exposure, whereby everyone receives the same number of infectious bites, always leads to the highest proportion infected (see Additional File 1). To understand this result, consider the problem from the hypothetical point of view of a mosquito population trying to infect as many humans as possible with malaria. In order not to waste any bites on people already infected, the mosquitoes should evenly distribute their bites on the entire population. If there is heterogeneity in the bite distribution, then many bites will be wasted on humans that are already infected.

### Heterogeneity in vaccine response

Leaky and all-or-nothing vaccines represent the extreme cases of a vaccine that has a completely homogeneous effect on the vaccinated population and a vaccine that has a very heterogeneous effect. In reality one would expect to observe a range of intermediate behaviours between these two extremes. A vaccine that has variable individual efficacy on the vaccinated population can be described by an efficacy distribution *g*. Let *g*(*x*) be the proportion of the population on which the vaccine has individual efficacy *x*. These efficacy distributions have been described previously by Halloran and Longini using frailty-mixing models [26,27]. Some examples are given in Table 3.

**Table 3:** Examples of distributions describing vaccine response in order of increasing heterogeneity.

Vaccine	Description
leaky	Vaccination gives everyone the same level of partial protection.
leaky-or-nothing	Vaccination offers partial protection to some people but no protection to others, as described by Halloran *et al *[27].
beta	Vaccination offers a variable level of protection to all vaccinees. The effect of vaccination follows a beta distribution as described by Maire *et al *[21].
all-or-nothing	Vaccination offers full protection to some people but no protection to others.

For a vaccine with efficacy distribution *g*, the average individual efficacy can be calculated by averaging the individual efficacy for the entire population

For a population in an area with force of infection Λ, given a homogeneous (leaky) vaccine with individual efficacy *VE*, the average proportion infected after follow-up time *T *will be *I*(*T*) = 1-*e*^-(1-*VE*)Λ*T*^. If the same population is instead given a vaccine with the same average individual efficacy *VE*, but with vaccine response described by a distribution *g *the average proportion infected will be

Figure 1B shows the average infected proportion as a function of the follow up time *T *for the efficacy distributions listed in Table 3 for a specified average individual efficacy. The all-or-nothing vaccine always protects more people from infection than the leaky vaccine regardless of follow-up time. Thus, the more heterogeneous a vaccine is (i.e. the more it resembles an all-or-nothing vaccine), the more people will be protected from infection. In fact it can be proved (see Additional File 1) that if a vaccine has average individual efficacy *VE*, then it will always protect at least as many people as would be expected from a leaky vaccine with the same average individual efficacy, and at most as many people as an all-or-nothing vaccine. To understand this, compare a leaky vaccine with an all-or-nothing vaccine. Given long follow-up, everyone in a group given a leaky vaccine will eventually become infected as eventually one of the infectious bites will evade the vaccine response. In contrast, a group given an all-or-nothing vaccine will contain a subgroup that will always remain free from infection. The unprotected subgroup will become infected at a natural rate, but the number of infections in this subgroup will be less than the number of infections in the entire leaky group. Therefore there will be fewer infections in the entire all-or-nothing group than in the leaky group.

### How heterogeneity affects estimates of vaccine efficacy

**Figure 2:**
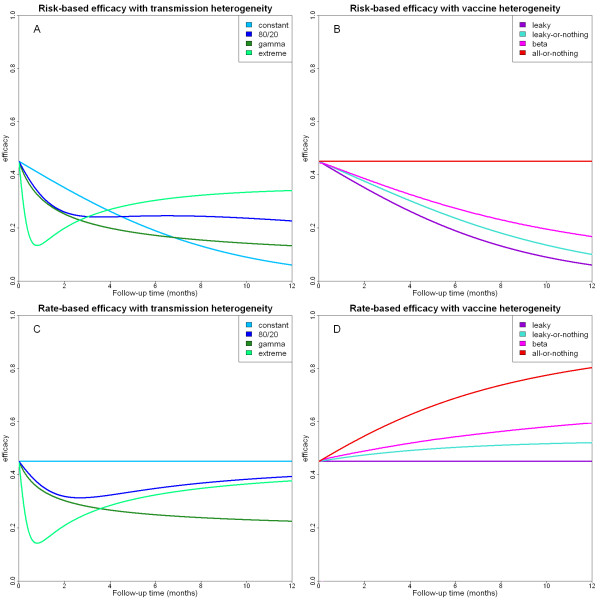
**(A) Risk-based infection-blocking efficacy for a vaccine under the range of transmission heterogeneities in Table 2**. (B) Risk-based infection-blocking efficacy for the four vaccines described in Table 3 with the same individual efficacy. (C) Rate-based infection-blocking efficacy under different transmission heterogeneities. (D) Rate-based infection-blocking efficacy for the four vaccines.

Consider an infection-blocking vaccine with constant individual efficacy being tested in a range of transmission settings described by the distributions in Table 2. Figures 2A and 2C show the risk-based and rate-based efficacies that would be observed as a function of increasing follow-up time. Comparing the curves for the risk-based (Figure 2A) and rate-based (Figure 2C) efficacies it is apparent that the rate-based efficacy is a more useful measure as it converges to the individual efficacy as the follow-up time increases. The estimates of rate-based efficacy in the presence of transmission heterogeneity (Figure 2C), show that the more heterogeneous the transmission setting, the lower the measured value for the rate-based infection-blocking efficacy. This means that if a vaccine is tested in an area with a high level of transmission heterogeneity, its efficacy will be underestimated.

To understand this, consider a population where 20% of the population receive 80% of the infectious mosquito bites as suggested by Woolhouse *et al *[28]. In the unvaccinated control group, the high-risk 20% will almost certainly become infected whereas only a small proportion of the low-risk 80% will become infected. Now consider the vaccinated group. Despite being vaccinated a large proportion of the high-risk 20% will develop malaria infection, as they will be bitten so frequently that the vaccine will be unable to block all infections. In the low-risk group, only a small proportion will become infected due to the low number of infectious bites and the effect of the vaccine. Thus, heterogeneity in the force of infection makes the infection profiles (who ends up infected) of the vaccine and control groups more similar, and hence a lower efficacy is measured.

Next consider an infection-blocking vaccine whose individual efficacy has been estimated from Phase IIa trials, but whose distribution of responses between individuals is unknown. Figures 2B and 2D show the risk-based and rate-based efficacies that would be observed for each of the response distributions described in Table 3. Note how the risk-based efficacies in Figure 2B decrease with follow-up time. This is because eventually everybody will become infected regardless of vaccination status. The exception to this is the case of an all-or-nothing vaccine where the subgroup which is fully protected will always remain uninfected. In contrast the rate-based efficacies in Figure 2D increase with follow-up time. This is because individuals who receive a high level of protection from the vaccine will remain susceptible for a longer time and significantly increase the total person years at risk used in the rate-based efficacy calculation. Finally, we observe that vaccines that induce more heterogeneous responses between individuals are estimated to be more efficacious than their homogeneous counterparts (Figure 2D), despite both vaccines having the same average individual efficacy. In particular, the upper limit for the observed rate-based efficacy is given by an all-or-nothing vaccine and the lower limit is given by a leaky vaccine. Therefore in a trial of a candidate malaria vaccine, if there is heterogeneity in vaccine response and this is ignored in the analysis, the vaccine efficacy will be overestimated.

### Waning vaccine efficacy

RTS, S has been observed to induce long-lasting protection against malaria for up to 45 months [29,30]. For example in a Phase II trial of children in Mozambique, Alonso *et al *[5] recorded a rate-based vaccine efficacy against first clinical episode of malaria of 29.9% (95% CI, 11.0-44.8) in a six-month follow-up period. In an extended follow-up of the Mozambique trial Sacarlal *et al *[30] recorded a vaccine efficacy of 16.8% (95% CI, -2.5-32.4) over months 21-33, and an efficacy of 11.8% (95% CI, -20.1-35.2) over months 33-45. These results could either be consistent with RTS, S inducing protection from infection over an extended period but with waning efficacy, or an artefact due to the apparent waning of efficacy due to the effects of heterogeneity.

Kanaan and Farrington [31] propose two models of waning vaccine efficacy; the selection and deterioration models. In the selection model, waning arises from heterogeneity in the duration of protection, i.e. a vaccinated person is protected for a period of time dependent on the vaccine half-life and then loses all protection. For example a person receiving a vaccine with initial individual efficacy 45% and a half-life of 1 year will have a 50% chance of still being protected after one year. In the deterioration model, waning arises from the gradual decline in the individual efficacy. For example a vaccine with initial individual efficacy of 45% and a half-life of 1 year will have an efficacy of 22.5% one year after it is administered. These two models are with-waning analogues of the all-or-nothing and leaky models. In both cases there can be additional heterogeneity between individuals in the rate of waning, with some individuals remaining protected for longer than others. This would occur if a vaccine's efficacy is dependent on natural boosting through continuous exposure to infection.

**Figure 3:**
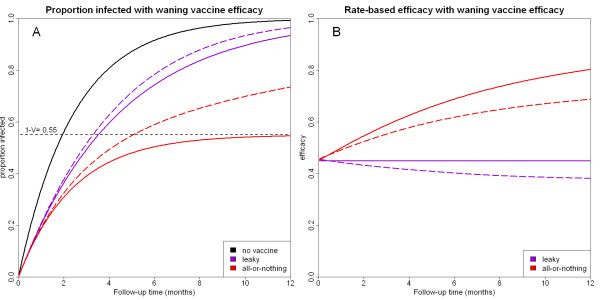
**(A) Cumulative proportion of vaccinated trial participants infected for leaky and all-or-nothing vaccines**. The dashed lines represent the infected proportion when vaccine efficacy wanes with a half-life of 1 year. (B) Rate-based efficacy for leaky and all-or-nothing vaccines. The dashed lines represent the expected rate-based efficacy with a waning vaccine.

Figure 3 shows the effects of waning efficacy for both leaky and all-or-nothing vaccines. The solid lines represent the cumulative infected proportion (Figure 3A) and the rate-based infection-blocking efficacy (Figure 3B) whilst the dashed lines represent these same quantities except with the addition of waning vaccine efficacy with a half-life of one year using the deterioration model for the leaky vaccine and the selection model for the all-or-nothing vaccine. As expected, waning efficacy reduces the observed rate-based vaccine efficacy for both leaky and all-or-nothing vaccines. If the effects of waning efficacy are ignored, estimates of rate-based efficacy can be prone to bias. For example, a waning vaccine with high initial efficacy could be confused with a non-waning vaccine with lower efficacy. Or a waning all-or-nothing vaccine could be mistaken for a non-waning leaky vaccine. Thus the only way to detect the presence of waning vaccine efficacy in clinical field trials is to ensure that there is extended follow-up of the trial participants.

## Discussion

The results presented here demonstrate that several sources of heterogeneity can lead to biased estimates of vaccine efficacy if their effects are ignored. In general, heterogeneity in exposure to infectious mosquitoes will result in an underestimate of vaccine efficacy. In contrast, heterogeneity in individuals' response to the vaccine (often termed the take of the vaccine) will result in an overestimate of vaccine efficacy. In addition, waning efficacy of the vaccine will further complicate the observed patterns. Thus simple calculations that ignore these sources of heterogeneity can easily give biased results and it will be difficult to assess the extent to which these are biased upwards or downwards.

Whilst these results are intuitively clear, it is often assumed that randomization will diminish or remove their effect. Clearly randomization should at the least ensure that characteristics such as individual-level attractiveness to mosquitoes are balanced in the trial arms. Moreover, cluster-based designs in which villages or other population units are randomized should ensure a balance in village-level exposure between the arms. However, even if heterogeneity is balanced across trial arms through robust randomization, this will not counter-balance the biases identified here. For exposure-driven heterogeneity, in the control arm those that are highly exposed will almost certainly become infected whilst the low-risk population will avoid infection. Similarly, in the vaccinated arm a large, albeit lower, proportion of the highly exposed population will become infected but as in the control arm, the remainder will likely avoid infection. Because a large proportion of both the control and vaccinated group are unexposed, this will always result in an underestimate of vaccine efficacy.

In contrast, heterogeneity in vaccine response will only affect the vaccine arm of the trial. In this arm of the trial heterogeneity will cause some trial participants to receive a higher level of protection than others, whereas the control arm will be unaffected. The presence of a highly protected subgroup in the vaccine arm of the trial will cause the measurement of the rate-based infection-blocking efficacy to be greater than the individual vaccine efficacy. Thus the individual vaccine efficacy will be overestimated.

Given these heterogeneities, how can their impact on estimates of vaccine efficacy be minimized? The first step is to ensure that trial participants are as homogeneous as possible. For example children in a malaria vaccine trial should be of similar age, live in similar settings and have similar access to healthcare and anti-malaria interventions such as bed nets. This should occur not just through randomization but by ensuring that trials are undertaken in areas of relatively homogeneous transmission. However even with the most carefully selected study cohort there is likely to be residual heterogeneity in exposure and response. Thus analysis methods need to account for these possible biases. In studies of malaria immunity Bejon [32] and Kinyanjui [33] have suggested that the effects of heterogeneity be reduced by excluding individuals likely to be unexposed. However, when infection rather than blood-stage disease is the primary outcome this may be problematic given that infection is generally the most reliable marker of exposure. In addition Valim *et al *[34] recently proposed a statistical estimator of individual vaccine efficacy that corrects for the effects of heterogeneity in exposure by taking account of multiple exposures. Whilst the authors are not aware of comparable estimators that account for heterogeneity in response, this may in fact not be a major problem for the RTS, S vaccine because in one of the Phase II trials of RTS, S [5], nearly all of the vaccinated group became infected with *P. falciparum *at some stage during the monitoring period. This suggests that the current RTS, S vaccine is leaky - although there may still be considerable heterogeneity in this leakiness. Thus further characterization of the heterogeneity in response is needed to ensure that estimates of vaccine efficacy are not substantially biased.

Extended follow-up of clinical trials of RTS, S have observed the apparent waning of vaccine efficacy against clinical malaria, as demonstrated by the coming together of Kaplan Meier plots comparing the infected proportions in vaccine and control groups [29]. This waning could be consistent with either a deterioration model where efficacy is gradually lost, or a selection model where efficacy is maintained for a random period of time dependent on the vaccine half-life, or may just be an artefact of heterogeneity in exposure and vaccine response. In addition there may be heterogeneity in waning where the vaccine has a variable half-life. Early efficacy estimates based on 6 or 12 month follow-up may be biased and this bias is difficult to quantify. For example, 12 month results could be consistent with a vaccine with high initial efficacy and a short half-life, or medium initial efficacy and a longer half-life. Extended follow-up, potentially spanning several years, is therefore required to obtain unbiased estimates.

## Conclusion

RTS, S is currently undergoing extensive testing in Phase III clinical trials. These trials will enrol up to 16,000 children and infants across 11 sites in seven different African countries. The results presented here demonstrate that heterogeneity in exposure, response and vaccine waning can bias vaccine efficacy measures in ways that are not easily measurable. This needs to be borne in mind when combining results from the wide range of transmission settings in these Phase III studies.

## Competing interests

ACG has received payment for advice on malaria transmission models provided to GSK.

## Authors' contributions

MTW and ACG conceived the study. MTW undertook the analysis with input from JTG, ACG and CJD. MTW, ACG, JTG and CJD prepared the manuscript.

## Supplementary Material

Additional file 1 **Mathematical Supplement**. Extended calculations and mathematical proofs. Click here for file
