# Supplementary material for: Heterogeneity in malaria exposure and vaccine response: implications for the interpretation of vaccine efficacy trials
Source: Malar J. 2010 Mar 23;9:82. doi: 10.1186/1475-2875-9-82 (PMC2851701; doi:10.1186/1475-2875-9-82)
Supplement: Additional file 1 — Mathematical Supplement. Extended calculations and mathematical proofs. [file 1475-2875-9-82-S1.DOC]

**Additional file 1: Mathematical Supplement**

**Calculating the expected infection proportion**

For an initially susceptible population in an area of constant, homogeneous malaria transmission with force of infection$\Lambda$, the expected infected proportion after time$T$,$I(T)$, can be described by the following simple equation

$$\begin{matrix} \frac{dI}{dt}=\Lambda(1-I) \\ I(0)=0 \end{matrix}$$

That is people in the susceptible class $S=1-I$ move to the infected class $I$at a constant rate$\Lambda$. This equation can be solved to give

$$I(T)=1-e^{-\Lambda T}$$

**Calculating the expected person years at risk**

In a trial, person years at risk are computed for each individual as the time from entry into the study to malaria infection, or to the end of the study period for those who do not become infected. For a single unvaccinated trial participant under conditions of homogeneous transmission intensity the expected person years at risk (PYAR) is calculated as

$$PYAR=\int_{0}^{T} e^{-\Lambda T}dt=\frac{1-e^{-\Lambda T}}{\Lambda}$$

When there is heterogeneity in transmission described by some distribution $f$ the mean PYAR can be calculated as follows

$$\begin{matrix} PYAR_{f}=\int_{0}^{T} \left(\int_{0}^{\infty} e^{-x\Lambda t}f(x)dx\right)dt \\ =\int_{0}^{\infty} \left[\frac{e^{-x\Lambda t}}{-\Lambda x}\right]_{0}^{T}f(x)dx \\ =\int_{0}^{\infty} \left(\frac{1-e^{-x\Lambda T}}{\Lambda x}\right)f(x)dx \end{matrix}$$

When there is heterogeneity in vaccine response described by some distribution $g$ the mean PYAR can be calculated as follows

$$\begin{matrix} PYAR_{g}=\int_{0}^{T} \left(\int_{0}^{1} e^{-(1-x)\Lambda t}g(x)dx\right)dt \\ =\int_{0}^{1} \left[\frac{e^{-(1-x)\Lambda t}}{-(1-x)\Lambda}\right]_{0}^{T}g(x)dx \\ =\int_{0}^{1} \left(\frac{1-e^{-(1-x)\Lambda T}}{(1-x)\Lambda}\right)g(x)dx \end{matrix}$$

**Table of transmission heterogeneity distributions with calculated heterogeneities**

A measure for the heterogeneity of a transmission distribution$f$, where $f$ has mean 1, can be obtained by calculating the variance of the distribution.

$$\begin{matrix} \sigma_{f}=\int_{0}^{\infty} (x-1)^{2}f(x)dx \\ =\int_{0}^{\infty} x^{2}f(x)dx-1 \end{matrix}$$

| **Transmission** | **Description** | **Distribution** | **Heterogeneity -**$\sigma_{f}^{2}$ |
| --- | --- | --- | --- |
| constant | All individuals receive the same number of infectious mosquito bites. | $\delta(x-1)$ | 0 |
| 80/20 | The distribution of infectious bites follows an 80/20 rule as suggested by Woolhouse *et al.* [28] where 20% of people receive 80% of the bites. | $\begin{matrix} 0.8\delta(x-0.25)+ \\ 0.2\delta(x-4) \end{matrix}$ | 3.25 |
| gamma | The distribution of infectious mosquito bites follows a gamma distribution with variance 4.2 as suggested by Smith *et al* [19]. |  | $4.2$ |
| extreme | A hypothetical example of extreme heterogeneity as might be observed in a localised epidemic, in this case modeled as 5% of people receiving 95% of infectious bites. |  | 18.053 |

**Table of vaccine response distributions with calculated heterogeneities**

A measure for the heterogeneity of a distribution of vaccine response$g$can be obtained by calculating the variance of the distribution

$$\begin{matrix} \sigma_{g}=\int_{0}^{1} (x-V)^{2}g(x)dx \\ =\int_{0}^{1} x^{2}g(x)dx-V^{2} \end{matrix}$$

| **Vaccine** | **Description** | **Distribution** | **Heterogeneity** -$\sigma_{g}^{2}$ |
| --- | --- | --- | --- |
| leaky | Vaccination gives everyone the same level of partial protection. | $\delta(x-V)$ | 0 |
| leaky-or-nothing | Vaccination offers partial protection to some people but no protection to others, as described by Halloran *et al.*[15] |  | $\frac{V^{2}}{3}$ |
| beta | Vaccination offers a variable level of protection to all vaccinees. The effect of vaccination follows a beta distribution as described by Maire *et al.* [21]. ($\beta=1$) |  | $\frac{V(1-V)^{2}}{\beta+1-V}$ |
| all-or-nothing | Vaccination offers full protection to some people but no protection to others. | $(1-V)\delta(x)+V\delta(x-1)$ | $V(1-V)$ |

**Proof that heterogeneity in transmission reduces the infected proportion in a clinical trial**

The heterogeneity in force of infection of malaria upon a population can be described by a distribution$f$. Let $F$be the family of such distributions, then $f\in F$satisfies

The expected cumulative proportion infected under a homogeneous force of infection will be $I(T)=1-e^{-\Delta T}$. Under a heterogeneous force of infection the expected infection proportion will be

$$I_{f}(T)=1-\int_{0}^{\infty} e^{-x\Lambda T}f(x)dx$$

To show that on average fewer people become infected in a heterogeneous setting, we need to prove $I_{f}(T)\leq I(T)$.

For $a=\Lambda T>0$prove

$$e^{-a}\leq\int_{0}^{\infty} e^{-ax}f(x)dx\leq1$$

First calculate the upper bound

$$U_{f}=\sup_{f\in F} \int_{0}^{\infty} e^{-ax}f(x)dx$$

Since $e^{-ax}\leq1\forall x\in[0,\infty)$we have

$$\int_{0}^{\infty} e^{-ax}f(x)dx\leq\int_{0}^{\infty} f(x)dx=1$$

so $U_{f}\leq1$. Now consider the family of mean-1 distributions

$$f_{\gamma}(x)=\frac{\gamma-1}{\gamma}\delta(x)+\frac{1}{\gamma}\delta(x-\gamma)$$

with

$$\int_{0}^{\infty} e^{-ax}f_{\gamma}(x)dx=\frac{\gamma-1}{\gamma}+\frac{1}{\gamma}e^{-\gamma a}$$

which tends to 1 as $\gamma\to\infty$. Thus

So $U_{f}=1$. Note that this bound is not attained in $F$since $\lim_{\gamma\to\infty} f_{\gamma}(x)=\delta(x)$which has mean 0, and therefore isn’t in $F$.

Now calculate the lower bound $L_{f}$.

$$L_{f}=\inf_{f\in F} \int_{0}^{\infty} e^{-ax}f(x)dx$$

Let $T(x)$be the tangent to $e^{-ax}$at $x=1$.

$$T(x)=e^{-a}(1+a-ax)$$

Since $T(x)\leq e^{-ax}\forall x$we have

$$\int_{0}^{\infty} e^{-ax}f(x)dx\geq\int_{0}^{\infty} T(x)f(x)dx=(1+a)e^{-a}\int_{0}^{\infty} f(x)dx-ae^{-a}\int_{0}^{\infty} xf(x)dx=e^{-a}$$

So $L_{f}\geq e^{-a}$. Now considering the mean-1 distribution $f(x)=\delta(x-1)$ we have

$$\int_{0}^{\infty} e^{-ax}\delta(x-1)dx=e^{-a}$$

so $L_{f}=e^{-a}$.

**Prove that a vaccine with individual vaccine efficacy V will protect at least as many people as a leaky vaccine and at most as many people as an all-or-nothing vaccine.**

The mean infected proportions for leaky and all-or-nothing vaccines with individual efficacy $V$will be given by

$$\begin{matrix} I_{\text{leak}}(T)=1-e^{-(1-V)\Lambda T} \\ I_{\text{aon}}(T)=(1-V)(1-e^{-\Lambda T}) \end{matrix}$$

The distribution of vaccine response for a vaccine with individual efficacy $V$can be described by a distribution$g$. Let $G$be the family of such distributions, then $g\in G$ satisfies

The mean infected proportion for a vaccine with response described by distribution $g$ will be

$$I_{g}(T)=1-\int_{0}^{1} e^{-(1-x)\Lambda T}g(x)dx$$

Prove that the mean proportion infected is always greater than it is for an all-or-nothing vaccine, and less than it is for a leaky vaccine, i.e.

$$I_{\text{aon}}(T)\leq I_{g}(T)\leq I_{\text{leak}}(T)$$

This is equivalent to proving

$$e^{aV}\leq\int_{0}^{1} e^{ax}g(x)dx\leq1+V(e^{a}-1)$$

for $a=\Lambda T>0$. First calculate the upper bound

$$U_{g}=\sup_{g\in G} \int_{0}^{1} e^{ax}g(x)dx$$

Let $U(x)$be the straight line between $(0,1)$and $(1,e^{a})$

$$U(x)=1+(e^{a}-1)x$$

Since $U(x)\geq e^{ax}\forall x\in[0,1]$ we have

$$\int_{0}^{1} e^{ax}g(x)dx\leq\int_{0}^{1} U(x)f(x)dx=\int_{0}^{1} g(x)dx+(e^{a}-1)\int_{0}^{1} xg(x)dx=1+V(e^{a}-1)$$

So $U_{g}\geq1+V(e^{a}-1)$. Now consider the function $g_{\text{aon}}(x)=(1-V)\delta(x)+V\delta(x-1)$ which gives

$$\int_{0}^{1} e^{ax}g_{\text{aon}}(x)dx=1+V(e^{a}-1)$$

And hence $U_{g}=1+V(e^{a}-1)$.

Now calculate the lower bound

$$L_{g}=\inf_{g\in G} \int_{0}^{1} e^{ax}g(x)dx$$

Let $L(x)$be the tangent to $e^{ax}$at the point $x=V$.

$$L(x)=e^{aV}(1-aV+ax)$$

Since $L(x)\leq e^{ax}\forall x\in[0,1]$ we have

$$\int_{0}^{1} e^{ax}g(x)dx\geq\int_{0}^{1} L(x)g(x)dx=(1-aV)e^{aV}\int_{0}^{1} g(x)dx+ae^{aV}\int_{0}^{1} xg(x)dx=(1-aV)e^{aV}+ae^{aV}V=e^{aV}$$

So $L_{g}\leq e^{aV}$. Now consider the function $g_{\text{leak}}(x)=\delta(x-V)$which gives

$$\int_{0}^{1} e^{ax}g_{\text{leak}}(x)dx=e^{aV}$$

and hence $L_{g}=e^{aV}$.
